# Perinatal origins of chronic lung disease: mechanisms–prevention–therapy—sphingolipid metabolism and the genetic and perinatal origins of childhood asthma

**DOI:** 10.1186/s40348-021-00130-y

**Published:** 2021-12-20

**Authors:** Emily Wasserman, Stefan Worgall

**Affiliations:** 1 Department of Pediatrics, Weill Cornell Medicine, 525 East 68th Street, Box 225, New York, NY 10065 USA; 2 Drukier Institute for Children’s Health, Weill Cornell Medicine, 413 East 69th Street, 12th Floor, New York, NY 10021 USA; 3 Department of Genetic Medicine, Weill Cornell Medicine, 1305 York Avenue, 13th Floor, New York, NY 10065 USA

**Keywords:** Sphingolipids, Asthma, 17q21, Serine-palmitoyl transferase, Perinatal, Microbiome, Rhinovirus

## Abstract

Childhood asthma derives from complex host-environment interactions occurring in the perinatal and infant period, a critical time for lung development. Sphingolipids are bioactive molecules consistently implicated in the pathogenesis of childhood asthma. Genome wide association studies (GWAS) initially identified a link between alleles within the 17q21 asthma-susceptibility locus, childhood asthma, and overexpression of the ORMDL sphingolipid biosynthesis regulator 3 (ORMDL3), an inhibitor of de novo sphingolipid synthesis. Subsequent studies of pediatric asthma offer strong evidence that these asthma-risk alleles correlate with early-life aberrancies of sphingolipid homeostasis and asthma. Relationships between sphingolipid metabolism and asthma-related risk factors, including maternal obesity and respiratory viral infections, are currently under investigation. This review will summarize how these perinatal and early life exposures can synergize with 17q21 asthma risk alleles to exacerbate disruptions of sphingolipid homeostasis and drive asthma pathogenesis.

## Introduction

Asthma is a heterogenous, chronic condition clinically identified by episodic shortness of breath, wheeze, and sometimes cough [1]. Once thought of as a single disease, asthma is now recognized as a spectrum of immunopathology culminating in a final common pathway of chronic airway inflammation, reversible airway obstruction, increased mucus production, and airway hyperreactivity. Globally, over 300 million people are affected by asthma and though the disease can occur at any age, it most often develops in childhood [2, 3]. Globally, asthma is a leading cause of childhood chronic illness [4]. The epidemiologic burden, which traditionally fell to metropolitan areas in high-income countries, is now increasing in low-income countries who also shoulder a disproportionate amount of asthma-related morbidity and mortality [2]. Available interventions to both prevent and treat severe asthma require frequent and expensive interactions with the health care system which limit school participation, work productivity, and overall quality of life [5]. Children with severe asthma are more likely to have symptoms persist through adulthood. The risk of adult-onset chronic obstructive pulmonary disease (COPD) is strongly associated with childhood deficiencies in lung function, measured by spirometry [2, 6, 7]. It remains unclear if the trajectory connecting childhood asthma and long-term respiratory morbidity can be reversed [8]. However, there is mounting evidence that exposures in the peri-natal and infant period serve as priming events for abnormal lung growth and lung inflammation, reflecting a possible avenue for childhood asthma prevention.

Classification systems for pediatric asthma have evolved significantly, and with them, the spectrum of asthma investigation. Chronic airway inflammation was previously considered the foundation of the two other key disease features, airway hyperresponsiveness and airway remodeling. Previous characterizations focused on the presence or absence of atopic, T-helper type 2 (Th2) cell inflammation [9–12]. Briefly, the Th2 pathway begins with allergen stimulation of Th2 cytokines (IL-4, IL-5, IL-9, IL-13) which trigger IgE release by B cells, which together promote histamine and leukotriene release by mast cells and eosinophilic inflammation [13]. General dampening of this inflammatory response by steroids or therapies targeting specific mediators within this pathway, i.e., leukotriene inhibitors and anti-IgE, IL4, or monoclonal antibodies, have been greatly effective for some but also revealed a broad group of pediatric non-responders with seemingly non-atopic and/or non-steroid responsive disease [14, 15].

**Fig. 1:**
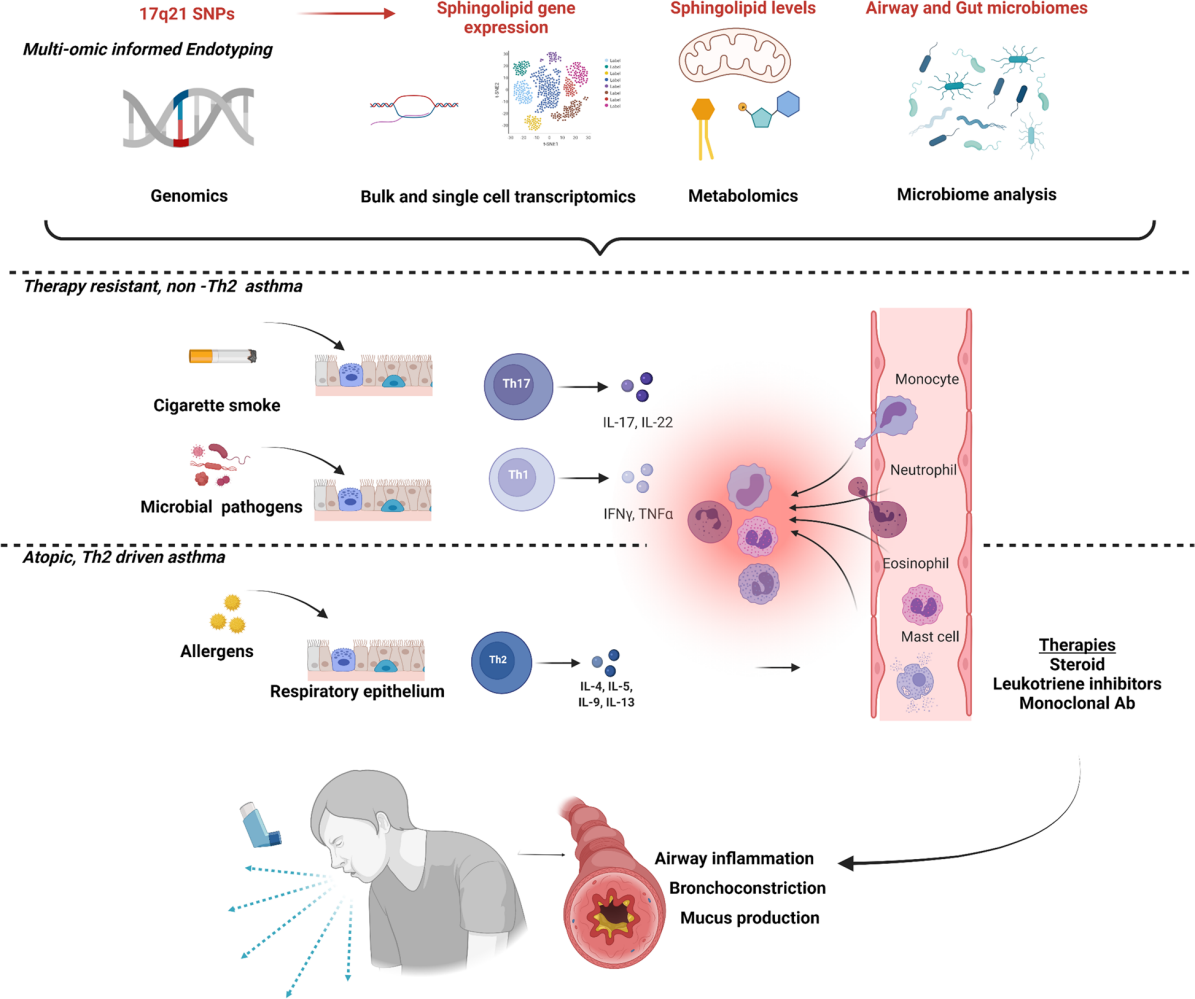
Expanding landscape of pediatric asthma investigation. Clinical features of wheeze, breathlessness, and cough were first related to the triad of airway inflammation, bronchoconstriction, and mucus production, followed by increased understanding of T-helper (Th) cell-associated pathways linking allergen exposure and Th2 cell cytokines to airway eosinophilia and mast cell degranulation. Neutrophil dominant airway inflammation was also identified and related Th1 and Th17 pathways, mediated by interferon γ and IL-17, respectively. Applied to samples from large pediatric asthma cohorts, technologies based on high throughput sequencing and mass spectrometry have revealed surprising, but correlative, genomic, and metabolic disturbances in connection with constitutive elements of the multiple inflammatory pathways. This includes the association of 17q21 SNPs with alterations to sphingolipid gene expression and metabolism in children with non-atopic asthma.

The limitations of allergy-based asthma therapies have driven efforts to develop more personalized methods for disease monitoring and treatment, beginning with the characterization of disease “endotypes.” With the advancement of high throughput technologies evolved a comprehensive approach, including genetic, metabolic, molecular, and clinical characteristics, to define more granular endotypes (Fig. 1) [8, 10, 16, 17]. This led to the increased recognition of non-Th2 inflammatory pathways including, Th1 and Th17, and the complex regulation of cells and cell mediators traditionally considered Th2. A subgroup of children with asthma display airway eosinophilia without associated Th2 cytokines [18]. Sputum transcriptomics has linked this type of airway eosinophilia to gene signatures from metabolic, ubiquitination, and mitochondrial function pathways [19]. The full range of asthma endotypes is beyond the scope of this review and is described in detail elsewhere [15, 20–22]. Despite the application of multi-omic technologies and related advances in asthma classification and treatment algorithms, therapy-resistant phenotypes persist, and early-life therapies have yet to change the long-term disease trajectory. Given the significant implications of childhood asthma on life-long respiratory health, there is an urgent need to address the origins of childhood disease. It is within this framework that sphingolipid metabolism has become a topic of interest for a fresh look on pathogenesis and therapies of childhood asthma.

Sphingolipids are bioactive molecules increasingly recognized in lung inflammation and airway hyperreactivity. Besides asthma, sphingolipids have been implicated in a host of chronic pulmonary disorders including bronchopulmonary dysplasia, chronic obstructive pulmonary disease (COPD), and cystic fibrosis [4]. In the context of asthma, attention turned to sphingolipids after genome-wide association studies (GWAS) reproducibly associated childhood asthma, and early-life wheeze with single-nucleotide polymorphisms (SNPs) within the region of chromosome 17q21 and increased expression of the sphingolipid synthesis regulator ORMDL3 [23–25]. Since then, aberrations in sphingolipid metabolism and gene expression have been seen in pediatric asthma cohorts [26, 27]. Animal models and in vitro studies have connected sphingolipid metabolism to clinical features of asthma, including airway hyperreactivity [28, 29]. This review will summarize the principal findings supporting a pathway from genetic and perinatal disruptions of sphingolipid metabolism to childhood asthma.

### Genetic dysregulation of sphingolipid metabolism in childhood asthma

**Fig. 2:**
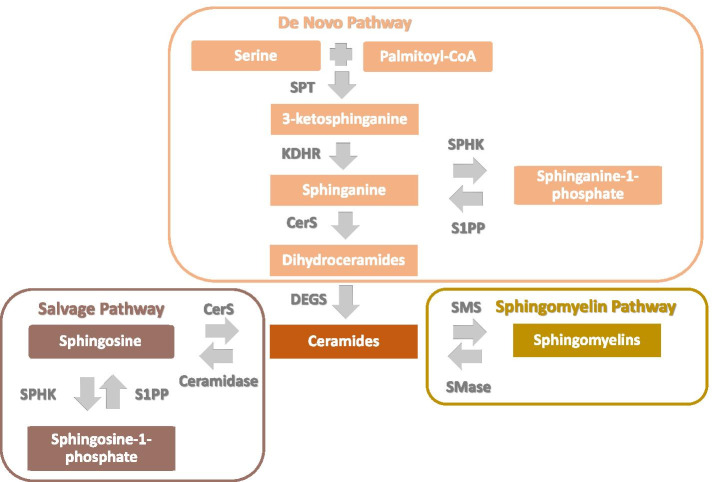
Pathways of Sphingolipid Metabolism. De novo synthesis begins with condensation of serine and palmitoyl CoA by serine palmitoyl-CoA transferase (SPT), the rate-limiting step in the production of 3-ketosphinganine, which is immediately reduced to sphinganine by ketodihydrosphingosine reductase (KDHR). Sphinganine can be phosphorylated by sphingosine kinases (SPHK) to sphinganine-1-phosphate or acylated by ceramide synthases (CERS) to form dihydroceramides. Dihydroceramides are converted to ceramides by dihydroceramide desaturase (DEGS), which are subsequently recycled as sphingosine, also by CERS, or converted to sphingomyelins by sphingomyelin synthase (SMS). Additional abbreviations SPP-1 (S1P phosphatase), and SMase (sphingomyelinase).

ORMDLs regulate de novo sphingolipid synthesis, which begins with the condensation of serine and palmitoyl CoA by serine palmitoyltransferase (SPT) in the endoplasmic reticulum (Fig. 2). In humans, ORMDL3 engages SPT, blocking its substrate pathway and suppressing its activity [30, 31]. GWAS studies showed ORMDL3 expression is increased with asthma risk alleles [23, 32], suggesting SPT inhibition is relevant to asthma pathogenesis. Though the genetic regulation of sphingolipid homeostasis is complex, and the mechanisms linking ORMDL3 to asthma are incompletely understood, there is mounting evidence that shifts in sphingolipid hemostasis have an important role in childhood asthma and early-life wheeze.

Sphingolipids are a ubiquitous and diverse class of amphipathic lipids comprised of a hydrophobic sphingoid base attached to a hydrophilic group which can consist of hydroxyl groups or, for more complex species, phosphates, and sugar residues [33]. Sphingolipids gain their complexity from the variable arrangement of these component parts. In mammalian cells, there are five known sphingoid bases with over twenty known arrangements of fatty acids, alkyl chain lengths, degrees of saturation, and hydroxylation. Sphinganine, the reduced product of the SPT catalyzed reaction, can be phosphorylated or deacylated to generate sphinganine-1-phosphate (Sa1P) or dihydroceramides, respectively. During the final step of the de novo synthesis, dihydroceramides are converted to ceramides. Ceramides, the nexus of sphingolipid metabolism, are the precursor to sphingomyelins, sphingosine, and sphingosine-1-phosphate (S1P) (Fig. 2) and more complex glycosphingolipids (not shown).

Studies measuring sphingolipid levels show consistent differences between children with asthma and children without asthma, though the relative direction of this difference varies by tissue compartment. Higher levels of ceramides and dihydroceramides were detected in exhaled breath condensates [34], serum [35], and plasma [27] of children with asthma. In a cohort of 5–17-year-old children, those with asthma displayed lower levels of sphingolipids in their blood cells. This finding was especially pronounced in children with non-allergic asthma. In this cohort, reduced blood sphingolipids are also associated with 17q21 asthma-associated risk alleles, specifically SNPs rs7216389 and rs8076131. Using heavy-isotope serine, metabolic labeling of the peripheral blood mononuclear cells from these children showed asthma and genotype-dependent decrease in de novo sphingolipid synthesis [27].

A study of two longitudinal mother-baby cohorts, the Copenhagen Prospective Study of Asthma in Childhood (COPSAC_2010_) and the Vitamin D Antenatal Asthma Reduction Trial (VDAART), also associated sphingolipid levels with early-life asthma. The study included plasma metabolomics at 6 months, 1 and 6 years, and transcriptomic analysis of nasal brushings at age 6. Interestingly, at age 6, asthma risk alleles were associated with reduced expression of the SPT subunits Sptlc1 and ssSPTa. The human SPT complex is composed of two large subunits, Sptlc1 and Sptlc2, and one small regulatory subunit, ssSPTa. This small subunit stabilizes the catalytic subunit Sptlc2 by altering its substrate specificity and greatly increases enzyme activity [31]. The association of the 17q21 risk alleles with lower expression of SPT subunits could point to an additional sphingolipid-regulatory mechanism associated with these genotypes. Relatedly, at age 6, there was also an inverse relationship between plasma Sa1P and airway resistance; and lower plasma Sa1P levels positively associated with 17q21 asthma risk alleles. Most interesting however is that the authors found a correlation between lower plasma sphingolipids (ceramides and sphingomyelins) at 6 months and the likelihood of asthma onset by age 3. The study clearly associates childhood asthma, 17q21 asthma genotypes with de novo pathway, but additionally suggests that disruptions in sphingolipid synthesis are present in infancy and predictive of later disease [26].

Sphingolipids are an integral part of plasma membranes, where they form discreet domains responsible for cellular processes including protein trafficking, signal transduction, and virus budding [36]. Outside of the plasma membrane, sphingolipids also serve as signaling molecules for a host of pathways including apoptosis [37], cytoskeletal reorganization, and cellular inflammation [36, 38]. While clinical studies correlate childhood asthma with alterations in sphingolipid production, animal and in vitro studies offer more granular insight into specific sphingolipid-lung interactions.

Animal models have connected ORMDL3 and sphingolipids to clinical features of asthma. ORMDL3 overexpressing mice display increased airway reactivity and airway remodeling, including increased airway smooth muscle, subepithelial fibrosis, and mucus [34, 39, 40]. Similar effects are seen with targeted inhibition of de novo sphingolipid synthesis. Both SPT haploinsufficient mice and wild-type mice treated with the SPT inhibitor myriocin display increased airway reactivity in the absence of allergic sensitization and airway inflammation [9, 28], suggesting a negative effect of lower sphingolipid synthesis on airway smooth muscle cells.

Increased ORMDL3 expression, both in mice and human lung epithelial cells, is associated with increased ceramide levels [34]. The sphingolipid mediator S1P is one of the most extensively studied sphingolipids in asthma [33, 41]. S1P is generated from the phosphorylation of sphingosine by one of two sphingosine kinases (SphK1 and SphK2). S1P modulates an array of biological processes and functions as both, intracellular second messenger and extracellular ligand for five known G protein-coupled receptors, S1PR1-5, [8]. SphKs and S1PRs are ubiquitously expressed, including in bronchial epithelial and airway smooth muscle cells [42]. Both S1P and SphK are associated with key pathogenic features of asthma including airway smooth muscle cell hyperresponsiveness and lung inflammation [9, 43]. In mice, administration of exogenous S1P increased airway resistance, bronchial contraction, and recruitment of inflammatory cells, namely mast cells and eosinophils [44]. Both ceramides and S1P emerge from the recycling/salvage pathway of sphingolipid synthesis. There is evidence to suggest that inhibition of the de novo sphingolipid synthesis pathway results in a compensatory upregulation of the recycling/salvage pathways [45]. In context with the observations from clinical cohorts, there is strong evidence that childhood asthma can evolve from a complex, integrated disruption of sphingolipid hemostasis.

### External factors influencing sphingolipid homeostasis

17q21 asthma risk alleles alter sphingolipid synthesis gene expression, leaving these pathways vulnerable to further disruption. Extrinsic factors, separately related to asthma, can influence sphingolipid synthesis. This includes perturbations of the host microbiome, maternal diet and obesity, and respiratory viruses. In the presence of 17q21 risk alleles, these elements may synergize to become the “second hit” necessary to shift sphingolipid metabolism toward asthma pathogenesis.

#### Maternal obesity during pregnancy

The in utero period is a critical time in lung development with long-term consequences for respiratory disorders [46–51]. There is strong evidence to support a link between childhood asthma and maternal obesity during pregnancy [50–63]. Large cohorts of mother-child dyads have shown an association between maternal obesity and early life bronchodilator use [58], but not atopic eczema or hay fever [55], suggesting that maternal obesity confers a non-atopic asthma phenotype. Interestingly, the VDAART study also found a relationship between childhood asthma and maternal sphingolipids in the third trimester. The risk of asthma correlated positively with maternal blood sphingomyelins levels and was inverse with maternal blood Sa1P [64]. The relationship between maternal obesity and sphingolipids requires further investigation, as both are strongly linked to non-atopic childhood asthma and early life wheeze.

#### Host microbiome

Since the proposal of the “hygiene hypothesis” by David Strachan in 1989, there have been extensive efforts to determine the contribution of the host-microbiome to asthma pathogenesis and immune dysregulation. Strachan postulated that improved standards of living and hygiene followed the reduction in household infections and also, increased risk of allergy [65]. Lack of infection resulted in poorly developed mechanisms of immune regulation including an unchecked Th2 dominant response. Interestingly, the Protection against Allergy Study in Rural Environment (PASTURE) found that the 17q21 genotypes that provide a risk for the development of asthma in wheezing infants also allow for environmental protection to allergen exposure [66]. It is well documented that patients with asthma display a relative dysbiosis of their lung, nasopharyngeal, and gut microbiomes [67, 68], even before the onset of the disease [69, 70]. Multiple studies have correlated the bacterial profile of infant stool, including colonization with *Clostridium difficile* and *Escherichia coli* and low levels of *Bifidobacteria*, with asthma development [71–73]. In both humans and mouse models, shifts in the gut microbiota have been associated with alterations to immune cell composition [74] and inflammatory mediators [75]. The field has progressed beyond bacterial community characterization to mapping host-microbe interaction and with that the metabolic consequences of the bacterial dysbiosis, including altered sphingolipid metabolism [76].

Bacterial sphingolipid synthesis is limited to members of the *Bacteroidetes* and selected *Proteobacteria* species. These bacteria are abundant in the mammalian gut where they can engage in a metabolic cross-talk with the host [77]. Previous studies have demonstrated the immunomodulatory activity of *B. fragilis* derived polysaccharides stimulation of CD4+ T cells and correction of Th1/Th2 imbalances [78]. A recent study found *Bacteroides*-derived sphingolipids are both sensed and incorporated into gut epithelial cell sphingolipid pathways [77]. In the gut, these bacterial sphingolipids can drive the recruitment and proliferation of invariant natural killer cells [76], a subset of T cells linked to multiple models of asthma [79]. Importantly, a recent report from the Baby Biome study found Cesarean section and intrapartum antibiotic use can significantly reduce the presence of Bacteroides species in the infant fecal microbiome [80]. These findings connect the intrapartum environment to microbiome-derived disturbances of sphingolipid homeostasis with meaningful implications for asthma development.

The infant gut microbiota is sensitive to multiple perinatal and early life exposures including maternal obesity, mode of delivery, gestational age, systemic antibiotics, breast vs formula feeding, cigarette smoke, household members, and pets. The relative contribution of each is currently under investigation. Efforts to reconstitute with gut microbiome with probiotics supplementation of *Lactobacillus* and *Bifidobacterium* with some studies showing reduction of asthma severity and others showing no effect [81].

Recently, studies have also shown that changes in the airway microbiome are associated with bronchiolitis in infants and young children [82–84]. Bronchiolitis shares several features of asthma, including airway inflammation and wheezing. Metabolomic analysis of nasopharyngeal samples from a cohort of infants hospitalized with bronchiolitis found a correlation between severe disease and upregulation of sphingolipid metabolism. *Streptococcus*, a dominant genus in the airway of infants with bronchiolitis, is positively associated with ceramide (18:2/16:0) and sphingomyelin (16:1/16:0) [85]. In the case of bronchiolitis and asthma, it remains to be determined if changes to sphingolipid metabolism precede or follow changes to the microbiome. It is clear however that the airway microbiome and metabolome are altered in the setting of early-life lower respiratory disease.

#### Respiratory viruses

Viral pathogens are responsible for most acute asthma attacks. There is substantial evidence that common respiratory viruses are not only a source of asthma-related morbidity, but also critical to disease inception. Infection with respiratory syncytial viral (RSV) [86] or human rhinovirus (HRV) [87] in the first 3 years of life significantly increases the risk of asthma later in childhood [88]. Epidemiologic studies have unmasked temporal relationships between early life viral infection and later allergen sensitization. Animal models have further revealed enhanced allergen sensitization and allergic airway inflammation following infection with influenza [89], RSV and HRV [90].

Both RSV and HRV interact with the sphingolipids during infection. RSV utilizes ganglioside GM1 in the assembly and release of viral particles [91]. RSV can also stimulate neutral ceramidase and SphK1 in lung epithelial cells prolonging their survival and in term, viral infection [92]. GWAS studies found early-life RV illness significantly strengthened the relationship between 17q21 asthma risk alleles and childhood asthma [93], suggesting RV is an important catalyst in asthma development. In vitro studies have exposed interactions between RV and sphingolipid synthesis. RV infection increases ceramide sphingolipids in epithelial cells [94]. Silencing of ORMDL3 in airway epithelial cells increases de novo sphingolipid synthesis and decreases expression of ICAM-1, the receptor for the majority of RV strains [95]. Inhibiting SPT also increases epithelial cell ICAM-1 expression [95] and RV replication [96]. These studies suggest genetic dampening of SPT activity may augment cellular responses to RV, allowing viral infection to further disrupt sphingolipid synthesis.

### Therapeutic manipulation of the sphingolipid pathway

Collectively, these studies suggest infancy and the perinatal period represent a vulnerable time for children with 17q21 asthma risk alleles. The maternal metabolome, with its many influences, along with common intrapartum and early-life exposures can irrecoverably offset their suboptimal sphingolipid homeostasis. The question that emerges is if the sphingolipid synthesis pathway can serve as a novel therapeutic target for both, prevention, and treatment of childhood asthma. Pharmacologic modification of sphingolipid metabolism in mice can attenuate asthma symptoms. Intranasal administration of FTY720, a structural analog of sphingosine, which can be phosphorylated by SphK and then act as an antagonist for S1PRs, reduces airway inflammation and hyperreactivity [34]. Similar effects can be elicited by inhibition of SphK1 [97]. To overcome the effects of decreased sphingolipid de novo synthesis a recent study trialed fenretinide, a dihydroceramide desaturase inhibitor that indirectly stimulates the de novo pathway, and GlyH-101, a chloride channel blocker that increases levels of multiple sphingolipids by an unknown mechanism [29, 98]. Both agents increased de novo sphingolipid metabolites in lung epithelial cells and reduced agonist-induced contraction in proximal and peripheral airways [29]. These studies suggest pharmacologic both induction of the de novo pathway and antagonizing some effects S1P are viable options for mitigating airway hyperreactivity.

## Conclusion

**Fig. 3:**
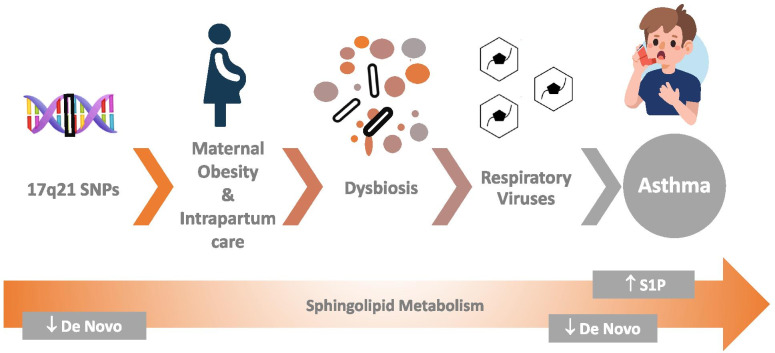
Schematic model for the genetic and extrinsic exposures affecting sphingolipid-driven asthma pathogenesis in children. 17q21 asthma risk alleles induce suboptimal de novo sphingolipid synthesis, which is exacerbated by perinatal and early life exposures including maternal obesity, mode of delivery, and intrapartum care, alterations to the host microbiome and respiratory viral infections. Together, these factors appear to tip the homeostatic balance away from the protective effects of a normal sphingolipid synthesis and toward the pathogenic effects of decreased de novo synthesis and increased S1P. Pregnant woman illustration author Sudowoodo, title "Bathroom and medical people icons stock illustration, USA, icon, people, women, men." Child with inhaler illustration author Irina_Strelnikova title "Asthma stock illustration: Asthmatic, Child, Inhaling, Illness, Asthma Inhaler", both provided by istockphoto.com

Asthma is a major cause of morbidity for children around the world. Multi-omic analyses of large pediatric cohorts have exposed several connections between sphingolipids and asthma/early life wheeze. These suggest asthma evolves from dynamic shifts in sphingolipid homeostasis, beginning with 17q21 asthma risk alleles and advancing with critical perinatal exposures that exacerbate genetic disruptions of sphingolipid metabolism. Maternal factors including weight, diet, mode of delivery, and intrapartum antibiotic use can directly and indirectly, via the gut microbiome, alter sphingolipid production. Post-partum, respiratory viral infections, and alterations of the airway microbiome can worsen these aberrations (Fig. 3). Together, these factors appear to tip the homeostatic balance toward lower de novo sphingolipid synthesis and increasing S1P.

Since the initial identification of 17q21 as an asthma risk region for childhood asthma, much attention has focused on how factors regulated and expressed in this region relate to the pathogenesis of childhood asthma. As a basic mechanism, genetically altered sphingolipid metabolism in children who are carriers of 17q21 asthma risk genotypes is thought to lead to functional effects on airway resistance and may act as a predisposing factor for the development of asthma. Study results in recent years suggest a strong association of 17q SNPs with the phenotype of persistent and intermediate wheezing in childhood, but not to allergic disease. It is possible that a specific form of childhood asthma exists that is characterized by decreased sphingolipid concentrations associated with 17q21 gene variants. Animal models suggest direct pharmacologic manipulation of the sphingolipid pathway can reset this balance. More work is needed to understand the role of sphingolipids in childhood asthma, as means of both preventing and treating this common disease.

## Data Availability

Not applicable

## Abbreviations

COPD: Chronic obstructive pulmonary disease
GWAS: Genome-wide association studies
SNP: Single-nucleotide polymorphism
ORMDL3: ORMDL sphingolipid biosynthesis regulator 3
SPT: Serine palmitoyl transferase
Sa1P: Sphinganine-1-phosphate
S1P: Sphingosine-1-phosphate
COPSAC_2010_: Copenhagen Prospective Study of Asthma in Childhood
VDAART: Vitamin D Antenatal Asthma Reduction Trial
SphK: Sphingosine kinases
RV: Human rhinoviruses
